# Reconstructive surgery of the nasal pyramid

**Published:** 2012

**Authors:** E Lăţcan, D Ferechide, CR Popescu

**Affiliations:** * “Prain” Medical Center, Bucharest, Romania; ** “Carol Davila” University of Medicine and Pharmacy, Bucharest, Romania; *** ENT Department, Colţea Clinical Hospital, Bucharest, Romania

**Keywords:** reconstruction, rhino correction, rhinoplasty, heterografts, silicone

## Abstract

The authors present their personal experience regarding the reconstruction of the nasal pyramid on 150 cases, encompassing both children and adults during the period 2000-2011.

Reconstruction of the nasal pyramid, regardless of age, was performed in cases of dysmorphias due to congenital malformations, accidents and surgical treatments with great loss of substance (benign or malignant tumors).

There were used the classical methods of reconstruction meaning rhino correction and rhinoplasty. However we mainly focused on the use of heterografts (implantable silicone implants - endoprostheses), or on the reconstruction of the nasal pyramid entirely with elastomeric silicone (epitheses), fixed with adhesive, implants and titanium magnets. Silicone, as a synthetic material, is very well tolerated by the body, having been demonstrated that it is a good oxygen carrier. Moreover, both nasal silicone and titanium implants are well tolerated as well and, in addition, they have good aesthetic, functional and psychological results. Patients regain an almost normal appearance, having good family and social integration and improved quality life.

## Introduction

The nose – the nasal pyramid- is an essential anatomic element of the central region of the face, with a mainly aesthetic role, brain protection in case of accidents (traumatic shock attenuation) and functional according to its positioning at the beginning of the upper respiratory tract.

It has an important role in breathing, smelling, tasting and protection of the lower respiratory tract (cleans, moistens and warms inspired air) [**1**].

Generally, according to sex and age, an individual is more concerned with the aesthetic appearance than with the functional aspect. Hence, the multitude of surgical techniques that emerged and developed many years ago depended on the technology that has appeared, and on the improved instruments, equipment and on the doctor’s training. There is no special technique to achieve reconstruction of the nasal pyramid. Many reconstructions were performed after the anatomic appearance of each patient’s nose [**2**].

In accordance with his training, the doctor must choose the most suitable method for the treatment of the patient, regardless of what the patient thinks and wishes. He must also explain with the help of photographs, measurements or endoscopic examination, which is the best option in order to obtain the suitable aesthetic and functional results. The patient’s appearance and psychological implications are very important and must be applied according to the surgical specialty. The patient needs to be correctly informed and he must give his signature that he understood the procedures that will be applied and agrees with the method that would be applied by the doctor. The disadvantage that might occur is that is not always possible to achieve the aesthetic aspect imagined or desired by the patient and that the evolution and surgery results might be seen after about 6 months or 1 year. Moreover, several complications may occur, especially due to lack of doctor-patient collaboration such as the patient’s failure to follow the post-surgery guidelines for care and check-up [**3**].

## Nasal pyramid reconstruction methods

### Indications:

- congenital malformations;

- accidents;

- after major surgical interventions: benign or malignant tumors.

Nasal pyramid dysmorphia may exist either by the lack or by surplus of substance such as soft parts, cartilages, bones [**4**].

### Types of nasal pyramid reconstruction:

I) By loss substance, depending on the materials used:

1. Reconstruction with biological materials: bone or cartilage auto and homografts;

2. Reconstruction with inert materials: heterografts, acrylic, polyethylene or silicone external (epitheses) or internal prostheses (endoprostheses - implant) [**3**].

II) By surplus of substance:

A) Rhino correction:

- nasal dysmorphia due to acute or chronic trauma or congenital malformation;

- luxations and fractures (recent or old);

- mutilations.

B) Rhinoplasty – nasal hyperplasias – aesthetics.

Objectives:

- pyramid height reduction;

- shortening of the pyramid with possible lobe lifting;

- narrowing the pyramid [**5**].

## Material and method

Silicone, invented in 1970, together with titanium implants, are synthetic materials well tolerated by the body (silicone is even used as a cardiac valve – because it has been demonstrated that it is a good oxygen carrier). Silicone is used for replacement, through methods of prosthetic reconstruction, of various parts of the human body, in case of great loss of substance, which cannot be replaced by common surgical techniques [**6**].

Titanium implants – invented in 1950 by Prof. Branemark P.I. (Sweden), with a very high purity (about 99,75 %), was initially used until 1977 in orthopedics and dentistry, while in the beginning of 1977s it has been also used in prosthetic cranio-facial and body reconstruction, for fixing prostheses with the Branemark osseointegration method [**7**]. Furthermore, titanium implant is very well tolerated (even a lifetime), without being toxic or allergic. The process of osseointegration is a process of oxiodoreduction that leads to bone growth around the implant, after a period of about 3 to 6 months from the surgical fixation [**8**].

There are many methods and techniques, depending on the defect, doctor’s training and patient’s wishes. Nasal pyramid reconstruction can be performed under local or general anesthesia. There are traditional and new prosthetic surgical techniques that use silicone and fixation through osseointegration with titanium implants or magnets [**9**].

Traditional surgical techniques:

- incision, lifting;

- reduction of the osseocartilaginous height;

- paramedian osteotomy;

- smoothing of the osseous nasal crest;

- lateral osteotomy;

- shortening of the pyramid;

- reduction of the triangular cartilages height;

- suture of the interseptocolumellar incision;

- internal contention (anterior nasal packing);

- external contention – with splint and adhesive bandages [**3**].

Prosthetic reconstruction surgery using silicone and titanium osseointegrated implants:

### I) Silicone epitheses:

They are indicated in great losses of substance, when it is not possible to intervene by conventional surgical methods in congenital malformations, accidents and after major surgery (benign or malignant tumors) [**7**].

A specific technique similar to the dental one is used, obtaining prostheses with the shape, size, color and elasticity of normal tissues, and with a good anatomical integration in the respective region. These prostheses are light, aesthetic and well tolerated by the organism. They restore human face as close to normal as possible, improving the patient’s quality of life and increasing his self-confidence [**9**].

Their fixation can be made either with adhesive or titanium implants, or surgically, with general or local anesthesia. They ensure greater stability, are easier to care and more aesthetic.

### II) Silicone endoprostheses:

Indications in alar insufficiency or nasal dysmorphias with loss of substance, in congenital malformations, accidents, operations.

The technique used is similar to the dental one, but it uses a special type of implantable silicone after 29 days, well tolerated by the organism, and which can remain in the body for the whole life. In the defective area, a cavity is surgically created, where the silicone endoprostheses is introduced [**10**].

Postoperative evolution and prognostic are generally favorable with aesthetic, functional and psychic results for the patient.

Complications – multiple and different, both for the surgeon and for the patient, both in the case of classical methods of prosthetic rehabilitation using silicone and titanium implants [**11**].

Complications can be – early postoperative, in the first 7 to 10 days, or late postoperative – after several months or years.

**Immediate postoperative complications (early)**

1. General complications:

- shock – rare in prolonged and very traumatic surgery with major blood loss – quick resolution in the Department of Anesthesia and Intensive Care;

- postoperative resorption fever – insignificant in the first 3 days, but indicating an infection in the following 5-6 days – antibiotic treatment;

- nausea, vomiting dark blood, digested and swallowed during surgery, or intolerance to postoperative anesthetics and antibiotics;

- urine retention – it is reflex.

2. Local complications:

- bleeding, hematoma;

- eyelid, face and forehead edema;

- skin redness;

- skin incision.

3. Septic complications – rare because of the intra and postoperative antibiotics use:

- abscess;

- inflammation of the lacrimal sac;

- acute infection of the upper airway tract;

- periostitis [**3**];

- necrosis around the titanium implants with their rejection [**7**].

**Late postoperative complications** can be aesthetic or functional:

- vicious scars – physiologic, hypertrophic (keloid), atrophic (retractile), or pigmentation;

- septoturbinate synechiae – breathing disorders;

- immune response – prostheses or homografts allergies;

- lowering of the raised tip;

- nose widening;

- movement or curving of the cartilage graft;

- bone or cartilage resorption in homografts;

- perichondritis;

- nasal algae;

- nasal pyramid dephormities;

- pharyngeal mycosis;

- hypoesthesia, paresthesia in the operated region;

- neuralgia;

- psychiatric disorders;

- Anosmia [**2**].

## CASE PRESENTATION

A) Epithesis – accidents :

**CASE 1 : animal bite mutilation (rat)**

**Fig. 1:**
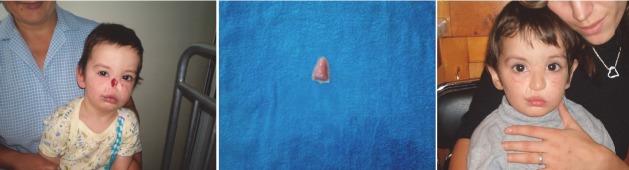
B.C. 1 year and 6 months, Bucharest. At the nursery, while he was sleeping, a rat ate his nose, mutilating him. Nasal pyramid was rebuilt using silicon prosthesis and fixed with adhesive. After the age of 6, titanium implants and magnets will be used. Aesthetic, social and functional results are very good.

**CASE 2 – animal bite mutilation (horse)**

**Fig. 2:**
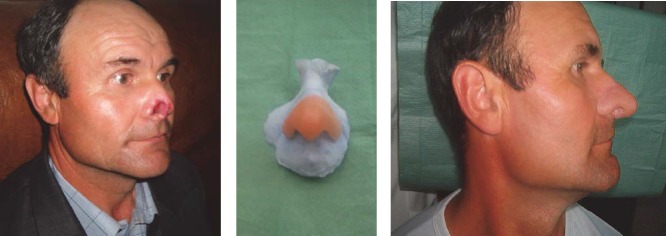
C.R. 42 years Teleorman, home accident with damage to the right nasal nostril because of a horse bite. Silicone Epitheses was built and fixed with adhesive. Favorable results.

**CASE 3 – malignant tumors surgery**

**Fig. 3:**
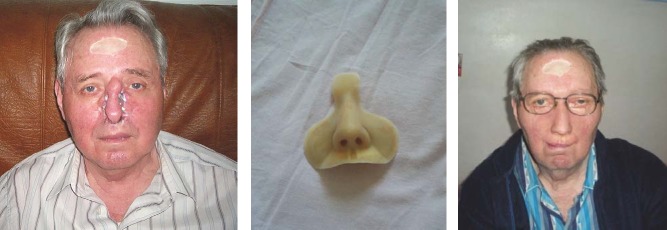
B.T. 63 years, Galati. Skin neoplasm of the nasal pyramid. Total rhinectomy was performed, and prosthetic reconstruction consisting in nasal pyramid silicon prosthesis fixed with adhesive was made. The patient regained his human appearance with improved breathing, sleep and increased quality of life.

**CASE 4**

**Fig. 4:**
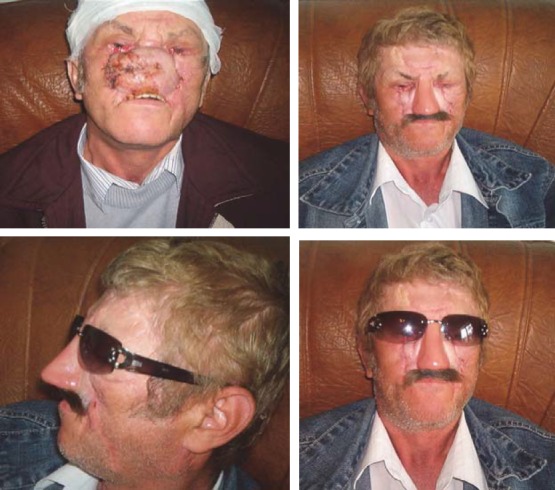
DV, 55 years old, Sibiu. Destruction of the nasal pyramid due to squamous cell carcinoma, which appeared after prolonged exposure to high temperature while working on the construction site in the Middle East. After a failed attempt of flap reconstruction in the Department of Plastic Surgery in Bucharest, a silicone nasal epithesis was made and fixed over the existing flap with adhesive. But the disease had a fulminate evolution with the invasion of sinuses, orbits, eyes, with brain metastases followed by the patient’s death after categorical refusal of surgical re-intervention or any other treatment.

**CASE 5**

**Fig. 5:**
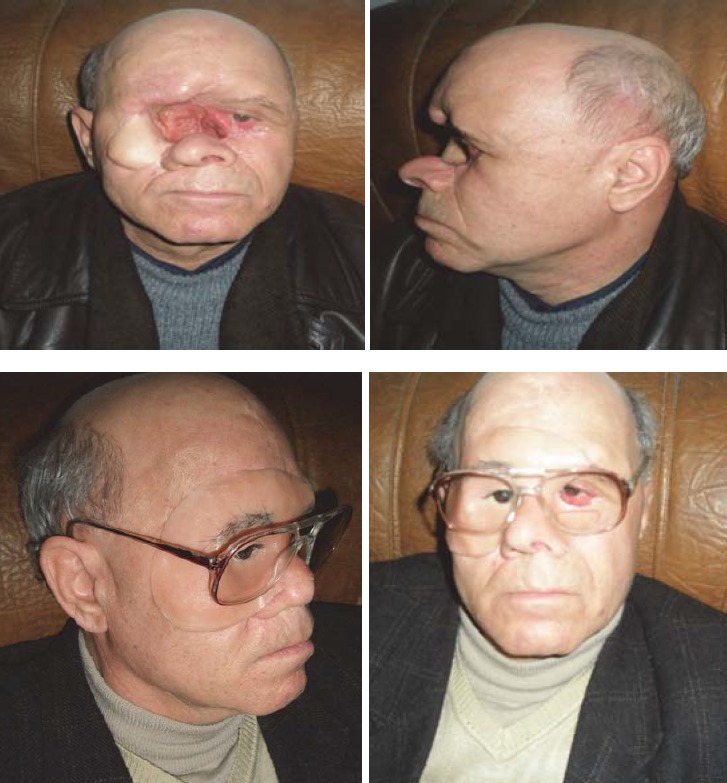
B.I., 68 years old, Bucharest, right maxillary sinus cancer diagnosed 10 years ago, with invasion of the right orbit and eye, of the nasal pyramid , with numerous surgeries in the Buco-Maxillofacial Department and Plastic Surgery Department in Bucharest. In 1999, the doctor suggested prosthetic reconstruction of the affected area (the half-upper right hemiface and eye orbit).In 2009, after unfavorable postoperative results, the patient required silicone reconstruction of almost 2/3 of his face.

**CASE 6**

**Fig. 6:**
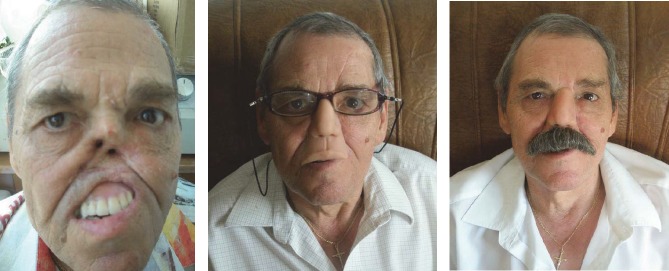
P.V., 65 years old, Bucharest-with total rhinectomy and resection of the first half of hard palate for squamous cell carcinoma. The nasal pyramid and upper lip were reconstructed with silicone, while the hard palate with insertion of an acrylic obturator prosthesis. Aesthetic and functional results were favorable, the prosthesis passing almost unnoticed if glasses and moustache are used.

**CASE 7**

**Fig. 7:**
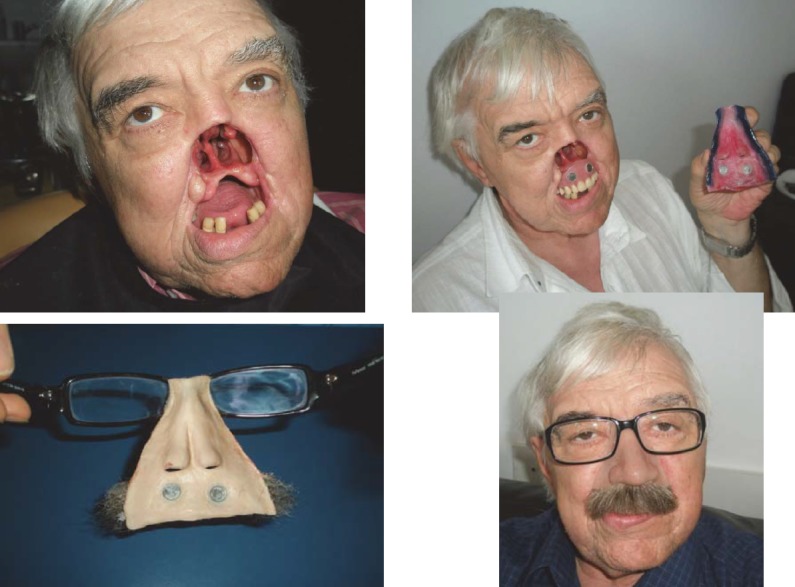
D.F, 64 years old, Bucharest. Total rhinectomy and left hemimaxillectomy with resection of the first half of the hard palate, surgery performed in UE for squamous cell carcinoma with left narinary starting point. The central part of the face was reconstructed with 2 extra oral silicon prosthesis with mustache (nose and upper lip epithesis) and one intraoral prosthesis (acrylic obturator with teeth). The prosthesis was fixed together with titanium minimagnets. The consolidation of the prosthetic assembly was made by fixing to progressive glasses, which provided the patient a safer travel environment. There were good aesthetic and functional results (breathing, chewing, eating, and speaking) and the patient presented good psychological condition.

B) Endoprosthesis-congenital malformation.

**1. Nasal valve insufficiency, lack of development of the bilateral alar cartilage during intrauterine life or acquired atrophy.**

**CASE 1**

**Fig. 8:**
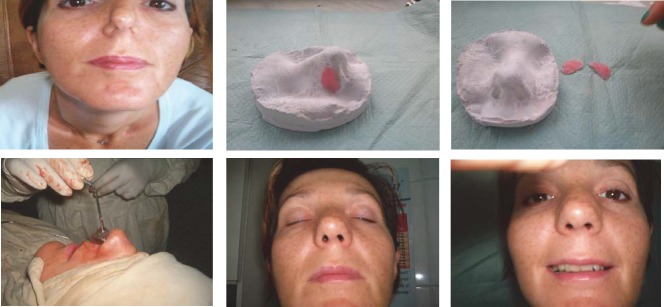
CV,, 38 years old Bucharest-chronic nasal obstruction with many treatments and surgery interventions. Implantable medical silicon splints are endonasally inserted with remedy of all functional deficiencies (breathing, sleep, mood, taste, smell). The patient presented good tolerance to silicon endoprosthesis without rejecting it so far (7 years).

**2. Congenital facial asymmetry with nasal dysmorphia-saddle nose.**

**CASE 2**

**Fig. 9:**
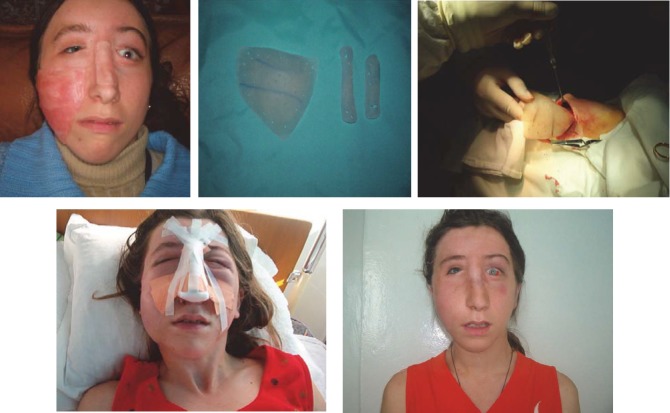
D.R.,19 years old, Bacau, facial asymmetry due to lack of development of the right hemi face dysmorphia with loss of substance (saddle nose). A complex reconstruction of the genian region, right cheek and dorsal region of the nose is performed using implantable silicone endoprosthesis, with good aesthetic results.

**3. Accidents**

a. Sledding

**CASE 1**

**Fig. 10:**
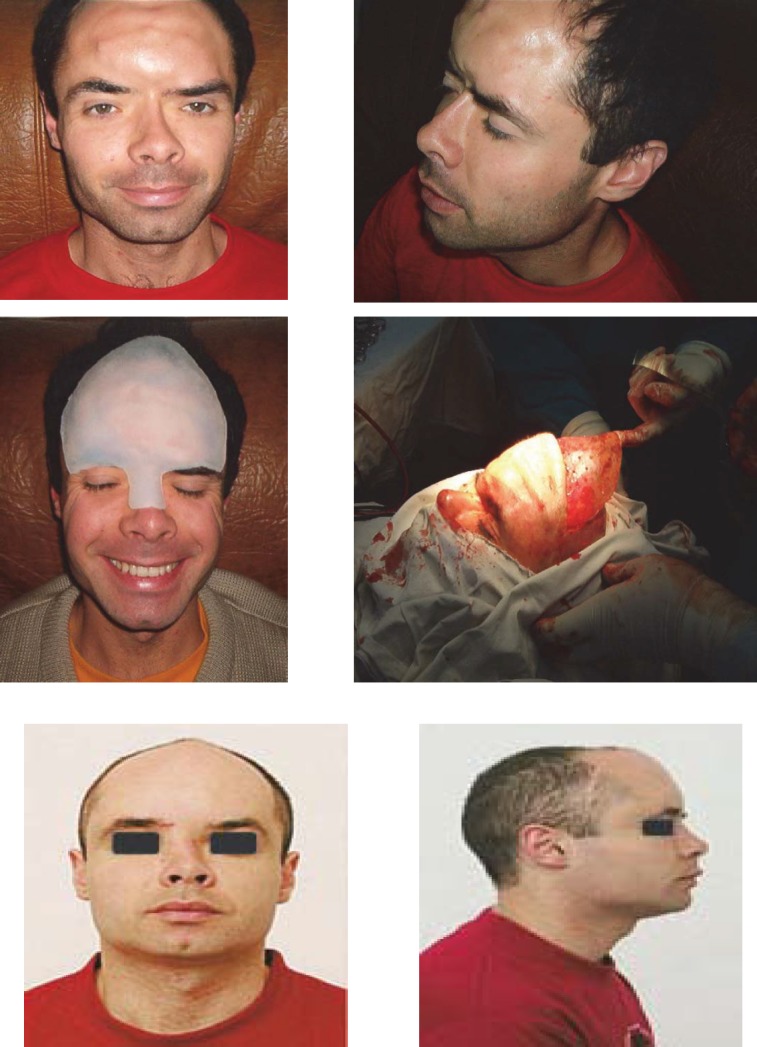
T.L., 34 years old, Brasov- sledding accident (impact of the skull with a tree trunk) with clogging of frontal region and the nose base, hypertelorism. The patient underwent repeated unsuccessfully attempts of aesthetic reconstruction of the frontal region with acrylate, by a neurosurgeon, and of nasal pyramid with an iliac crest graft, by an ENT specialist. After almost 10 years, a subcutaneous implantable silicone endoprosthesis was used for frontal region and nasal pyramid. Aesthetic and functional results were very good, the prosthesis fitting perfectly in the area by tissue growth around it. The patient is married now and has two children.

**b. Perinatal accident**

**CASE 2**

**Figure 11.**
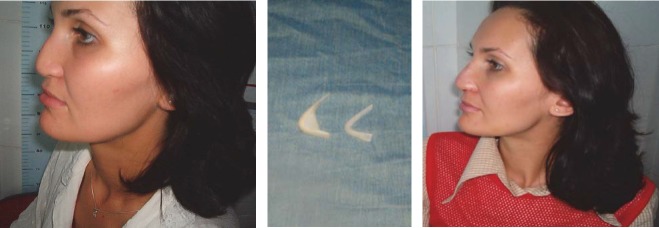
M.L., 26 years old, Teleorman - deformation of the nasal pyramid at birth, through obstetrical maneuvers. After numerous failed attempts, a surgical reconstruction with implantable silicone was performed.

**Conclusions**

- Surgical reconstruction of the nasal pyramid with the multitude of surgery techniques, despite accidents or postoperative complications, is a useful technique in the aesthetic and functional reconstruction of the central region of the face.
- It is worth mentioning the modern technique of surgical prosthetic reconstruction in cases of large loss of substance, when the common surgical procedures are inefficient.
- Silicone and titanium implants are well tolerated by the human body, without being toxic or allergic.
- Aesthetic role –the patient regains a normal appearance by restoring the normal anatomy of the respective area.
- Functional role-in breathing, mastication, phonation, hearing.
- Psychological implications in socialization and improvement of the quality of life.
